# Supplementary material for: Identification of an Immunogenic Medulloblastoma-Specific Fusion Involving EPC2 and GULP1
Source: Cancers (Basel). 2021 Nov 21;13(22):5838. doi: 10.3390/cancers13225838 (PMC8616194; doi:10.3390/cancers13225838)
Supplement: Supplementary file 1 [file cancers-13-05838-s001.zip › cancers-1461457-supplementary.pdf]

Supplementary Material

Identification of an Immunogenic Medulloblastoma-Specific Fusion Involving *EPC2* and *GULP1*

Claudia Paret, Nadine Lehmann, Hannah Bender, Maximilian Sprang, Clemens J. Sommer, Denis Cana, Larissa Seidmann, Arthur Wingerter, Marie A. Neu, Khalifa El Malki, Francesca Alt, Lea Roth, Federico Marini, Malte Ottenhausen, Martin Glaser, Markus Knuf, Alexandra Russo and Joerg Faber

A

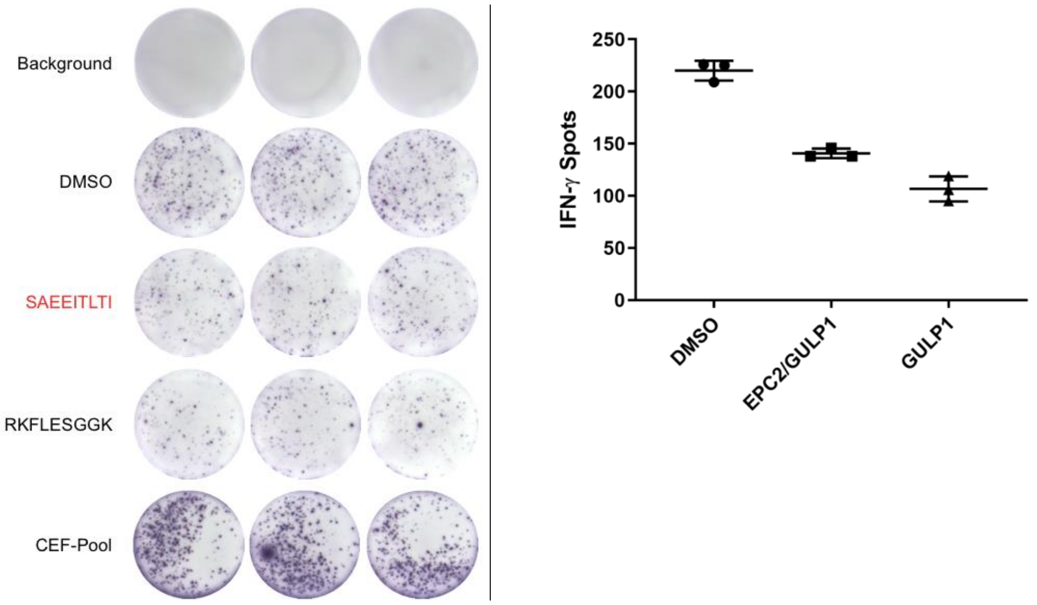

B

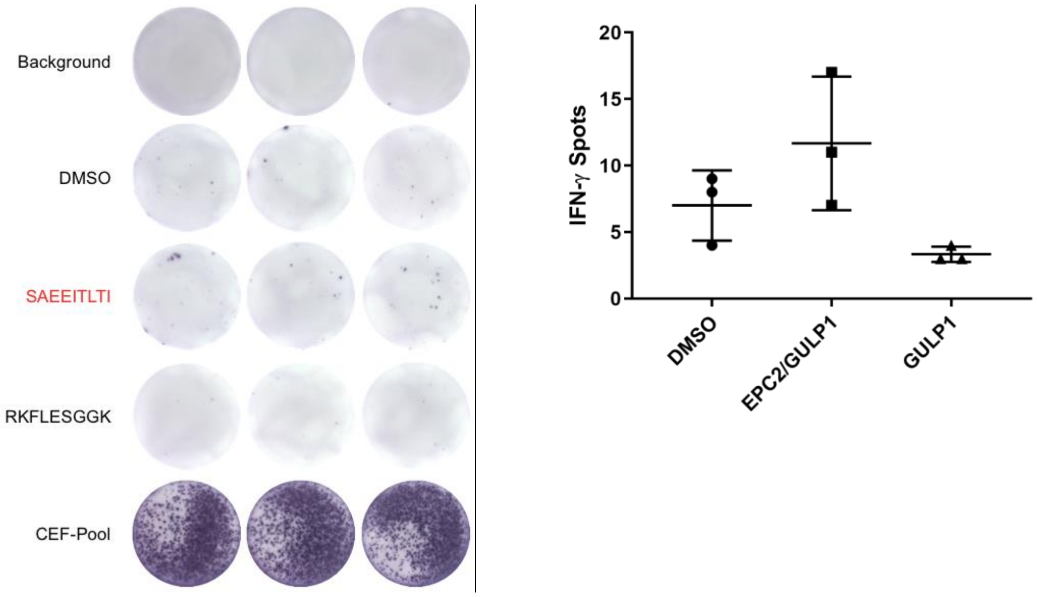

Figure S1: ELISpot analyses of healthy donor 2 and 3 show reactivity of CD8+ T cells towards fusion peptide ELISpot analysis after in vitro assay based on co-culturing peptide loaded DCs with CD8+ T cells of healthy

donor 2 and 3. DMSO was taken as vehicle control, wildtype peptide of GULP1 as negative control and CEF-Pool as positive control. Strong IFN-γ secretion of T cells after stimulation with the fusion peptide (red marked). **A**) Healthy donor 2: The IFN-γ secretion is 1.3-fold higher than after stimulation with wildtype peptide. There is also a strong reactivity towards the vehicle control DMSO. **B**) Healthy donor 3: The IFN-γ secretion is 3.5-fold higher than after stimulation with wildtype peptide.

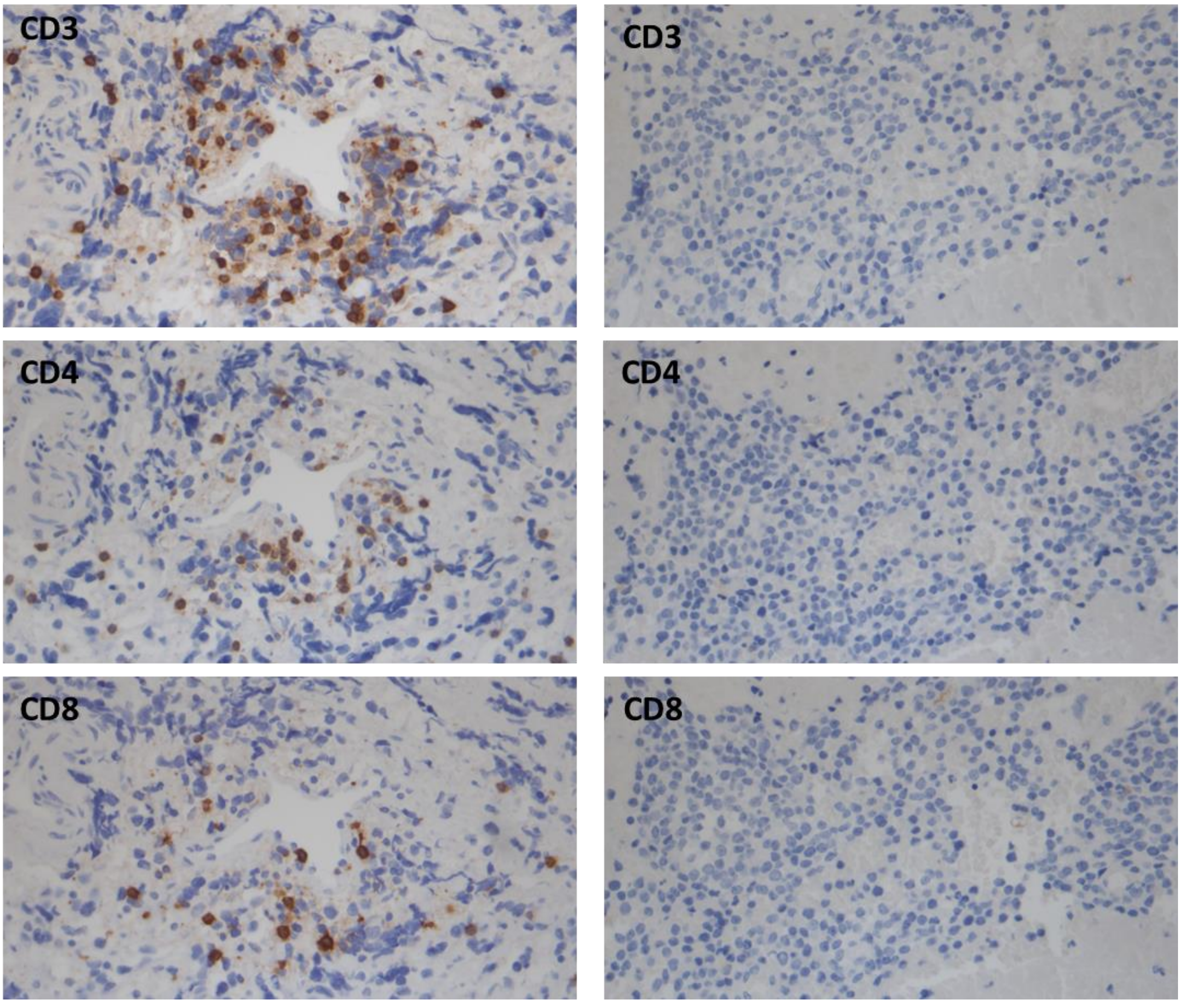

Figure S2. Infiltrating T cells in medulloblastoma Immunohistochemistry was performed on 3 µm thick routinely processed formalin-fixed and paraffin-embedded tissue sections. Sections derived from the tumor of patient no 25 (left panel, tumor positive for *EPC2-GULP1* fusion) or no 129 (right panel, tumor negative for *EPC2-GULP1* fusion) were stained with anti-CD3, CD8 and CD4 primary antibody. Magnification 400X.

Table S1: Summary of publications in the context of gene fusions analyses in medulloblastoma patients.

Publication	Summary
**Northcott et al. 2017**	Analysis of the somatic landscape across 491 sequenced medulloblastoma samples; 20 fusions across all medulloblastoma subtypes; Fusions involving medulloblastoma driver genes, e.g. *GLI2*, *PTEN*, *SUFU*; 8 in-frame fusions, 9 frame-shift fusions, 3 non-coding fusions

	no recurrent* gene fusions
**Luo et al. 2021**	Comparison of Asian and Caucasian cohort (Data source Caucasian cohort Northcott et al. 2017); Analysis of 52 tumor samples from Asian cohort, approx. 149 gene fusions events found; one recurrent* in-frame gene fusion *DNMT1-ZGLP1* (2/52) with identical breaking points only in the Asian cohort; 31 novel fusion genes from at least two samples in the Asian and Caucasian cohort
**Northcott et al. 2012**	Analysis of somatic copy number aberrations in 1,087 medulloblastoma samples; Fusion *PVT1-MYC* in group 3 medulloblastoma with *MYC*-amplification in 12 out of 60 samples (60%); *PVT1* is a non-coding host gene; all *PVT1-MYC* fusions with different breaking points, in some cases with additional compartments of chromosome 8
**Jones et al. 2012**	Analysis of 125 medulloblastoma samples; Identification of 3 gene fusions *DNAJB6-SHH* (SHH subgroup), *LCLAT1-ERBB4* Group 3), *MLLT6-MRPL45* (Group 4); no recurrent* gene fusions
**Skowron et al. 2021**	Cohort of 250 medulloblastoma samples (SHH subgroup); recurrent gene fusions affecting *GLI2, EPB41L5, NBAS, BCAS3, GLIS3, ZBTB20, SUFU, PTCH1* predominantly with different fusion partners; fusions with same gene partners have different breaking points; 3 recurrent* fusion transcripts with identical breaking points: SUCO-NCOR1 (2/250), *C4BPB-NCOR1* (3/250), *PAC6-GNAS* (2/250)

Table S2: Overview of other brain tumor samples analyzed in this work.

Sample	Age at diagnosis	Diagnosis	*EPC2-GULP1* fusion
**84**	8	Astrocytoma	no
**95**	10	Glioblastoma	no
**121**	14	Ependymoma	no
**122**	12	Astrocytoma	no
**123**	6	HGNET-BCOR	no
**139**	10	Glioblastoma	no
**143**	10	Astrocytoma	no

**159**	3	Astrocytoma	no
**226**	13	Astrocytoma	no
**281**	15	Astrocytoma	no
**294**	9	Ependymoma	no
**323**	11	Astrocytoma	no
**340**	3	Astrocytoma	no

Table S3: *In silico* epitope prediction with SYFPEITHI algorithm from selected, published in-frame fusion events. Peptide is predicted as a potential immunogenic epitope if its SYFPEITHI score is ≥ 20. Amino acids representing the fusion point are highlighted in bold letters.

**in-frame fusion (MB-Subtype)**	**AA-sequence**	**9mer peptides (HLA-haplotype; SYFPEITHI Score)**	**Reference**
TCF4-ROCK1 **(SHH)**	KVPPGLPS**SD**GLDALVYD*	S**SD**GLDALV (HLA-A*02:01; **17**)<br>PS**SD**GLDAL (HLA-A*02:01; **15**)<br>PGLPS**SD**GL (HLA-A*02:01; **12**)<br>GLPS**SD**GLD (HLA-A*02:01; **11**)<br>LPS**SD**GLDA (HLA-A*02:01; **7**)<br>VPPGLPS**SD** (HLA-A*02:01; **6**)<br>**SD**GLDALVY (HLA-A*02:01; **3**)<br>PPGLPS**SD**G (HLA-A*02:01; **-2**)	Northcott et al. 2017
PTEN-THAP9 **(Group 3)**	RNNIDDVV**RW**LSKCQPSP*	NIDDVV**RW**L (HLA-A*02:01; **23**)<br>NNIDDVV**RW** (HLA-A*02:01; **9**)<br>DVV**RW**LSKC (HLA-A*02:01; **9**)<br>VV**RW**LSKCQ (HLA-A*02:01; **5**)<br>**RW**LSKCQPS (HLA-A*02:01; **3**)<br>IDDVV**RW**LS (HLA-A*02:01; **2**)<br>DDVV**RW**LSK (HLA-A*02:01; **2**)<br>V**RW**LSKCQP (HLA-A*02:01; **2**)	Northcott et al. 2017
MARCKSL1-PIK3CD **(Group 3)**	ASPAKANG**QL**LWHRAQYE*	SPAKANG**QL** (HLA-A*02:01; **14**)<br>**QL**LWHRAQY (HLA-A*02:01; **14**)<br>KANG**QL**LWH (HLA-A*02:01; **13**)<br>PAKANG**QL**L (HLA-A*02:01; **10**)<br>AKANG**QL**LW (HLA-A*02:01; **7**)<br>ANG**QL**LWHR (HLA-A*02:01; **7**)<br>NG**QL**LWHRA (HLA-A*02:01; **6**)<br>G**QL**LWHRAQ (HLA-A*02:01; **4**)	Northcott et al. 2017

DNMT1-ZGLP1 (Group 4)**	SLPDDVRR**rS**LWPACQES	PDDVRR**RS**L (HLA-A*02:01; **9**)	Luo et al. 2021
		VRR**RS**LWPA (HLA-A*02:01; **9**)	
		DVRR**RS**LWP (HLA-A*02:01; **4**)	
		LPDDVRR**RS** (HLA-A*02:01; **3**)	
		DDVRR**RS**LW (HLA-A*02:01; **-1**)	
		DDVRR**RS**LW (HLA-A*24:02; **3**)	
		LPDDVRR**RS** (HLA-A*24:02; **2**)	
		**RS**LWPACQE (HLA-A*24:02; **2**)	
		VRR**RS**LWPA (HLA-A*24:02; **1**)	
		R**RS**LWPACQ (HLA-A*24:02; **1**)	
		DVRR**RS**LWP (HLA-A*24:02; **0**)	
		RR**RS**LWPAC (HLA-A*24:02; **-1**)	
NFYC-PPIE (SHH)**	IIAQPQQG**QK**QEESAITS	G**QK**QEESAI (HLA-A*02:01; **9**)	Luo et al. 2021
		IAQPQQG**QK** (HLA-A*02:01; **8**)	
		**QK**QEESAIT (HLA-A*02:01; **8**)	
		QG**QK**QEESA (HLA-A*02:01; **6**)	
		AQPQQG**QK**Q (HLA-A*02:01; **4**)	
		QQG**QK**QEES (HLA-A*02:01; **3**)	
		QPQQG**QK**QE (HLA-A*02:01; **1**)	
		PQQG**QK**QEE (HLA-A*02:01; **1**)	
		G**QK**QEESAI (HLA-A*24:02; **9**)	
		AQPQQG**QK**Q (HLA-A*24:02; **4**)	
		QQG**QK**QEES (HLA-A*24:02; **2**)	
		QG**QK**QEESA (HLA-A*24:02; **2**)	
		**QK**QEESAIT (HLA-A*24:02; **2**)	
		IAQPQQG**QK** (HLA-A*24:02; **1**)	
		QPQQG**QK**QE (HLA-A*24:02; **0**)	
		PQQG**QK**QEE (HLA-A*24:02; **0**)	
ANKRD52-CS (Group 3)**	RGRTALHR**GV**VPGYGHAV	ALHR**GV**VPG (HLA-A*02:01; **20**)	Luo et al. 2021
		RTALHR**GV**V (HLA-A*02:01; **16**)	
		**GV**VPGYGHA (HLA-A*02:01; **13**)	
		GRTALHR**GV** (HLA-A*02:01; **12**)	
		LHR**GV**VPGY (HLA-A*02:01; **10**)	
		TALHR**GV**VP (HLA-A*02:01; **8**)	
		R**GV**VPGYGH (HLA-A*02:01; **3**)	
		HR**GV**VPGYG (HLA-A*02:01; **0**)	
		TALHR**GV**VP (HLA-A*24:02; **4**)	
		HR**GV**VPGYG (HLA-A*24:02; **2**)	
		**GV**VPGYGHA (HLA-A*24:02; **2**)	
		GRTALHR**GV** (HLA-A*24:02; **1**)	
		RTALHR**GV**V (HLA-A*24:02; **1**)	
		LHR**GV**VPGY (HLA-A*24:02; **1**)	

		ALHR**GV**VPG (HLA-A*24:02; **0**)<br>R**GV**VPGYGH (HLA-A*24:02; **0**)	
ASAP1-WDYHV1* (Group 3)*	SSRDSLWN**r**EENIWKLCE	SLWN**RE**ENI (HLA-A*02:01; **21**)<br>N**RE**ENIWKL (HLA-A*02:01; **17**)<br>SRDSLWN**RE** (HLA-A*02:01; **4**)<br>LWN**RE**ENIW (HLA-A*02:01; **4**)<br>DSLWN**RE**EN (HLA-A*02:01; **3**)<br>RDSLWN**RE**E (HLA-A*02:01; **2**)<br>WN**RE**ENIWK (HLA-A*02:01; **2**)<br>**RE**ENIWKLC (HLA-A*02:01; **-2**)<br>NREENIWKL (HLA-A*24:02; **15**)<br>SLWNREENI (HLA-A*24:02; **12**)<br>DSLWNREEN (HLA-A*24:02; **3**)<br>REENIWKLC (HLA-A*24:02; **3**)<br>WNREENIWK (HLA-A*24:02; **1**)<br>SRDSLWNRE (HLA-A*24:02; **0**)<br>RDSLWNREE (HLA-A*24:02; **0**)<br>LWNREENIW (HLA-A*24:02; **0**)	Luo et al. 2021
SAE-NPAS1* (Group 4)*	TTSDYFLL**Qa**PGRRGPAA	FLL**QA**PGRR (HLA-A*02:01; **13**)<br>LL**QA**PGRRG (HLA-A*02:01; **13**)<br>**QA**PGRRGPA (HLA-A*02:01; **10**)<br>TSDYFLL**QA** (HLA-A*02:01; **9**)<br>L**QA**PGRRGP (HLA-A*02:01; **9**)<br>SDYFLL**QA**P (HLA-A*02:01; **8**)<br>YFLL**QA**PGR (HLA-A*02:01; **6**)<br>DYFLL**QA**PG (HLA-A*02:01; **3**)<br>DYFLL**QA**PG (HLA-A*24:02; **10**)<br>YFLL**QA**PGR (HLA-A*24:02; **8**)<br>FLL**QA**PGRR (HLA-A*24:02; **3**)<br>**QA**PGRRGPA (HLA-A*24:02; **3**)<br>TSDYFLL**QA** (HLA-A*24:02; **1**)<br>L**QA**PGRRGP (HLA-A*24:02; **1**)	Luo et al. 2021

		SDYFLL**QA**P (HLA-A*24:02; **0**)<br>LL**QA**PGRRG (HLA-A*24:02; **0**)	
TET3-GMCL1* (Group 4)***	SIRELMEE**rC**LEWLLNNL	LMEE**RC**LEW (HLA-A*02:01; **14**)<br>MEE**RC**LEWL (HLA-A*02:01; **13**)<br>RELMEE**RC**L (HLA-A*02:01; **12**)<br>ELMEE**RC**LE (HLA-A*02:01; **10**)<br>**RC**LEWLLNN (HLA-A*02:01; **9**)<br>EE**RC**LEWLL (HLA-A*02:01; **8**)<br>IRELMEE**RC** (HLA-A*02:01; **1**)<br>E**RC**LEWLLN (HLA-A*02:01; **-2**)<br>RELMEE**RC**L (HLA-A*24:02; **12**)<br>MEE**RC**LEWL (HLA-A*24:02; **11**)<br>EE**RC**LEWLL (HLA-A*24:02; **11**)<br>ELMEE**RC**LE (HLA-A*24:02; **4**)<br>**RC**LEWLLNN (HLA-A*24:02; **4**)<br>LMEE**RC**LEW (HLA-A*24:02; **3**)<br>E**RC**LEWLLN (HLA-A*24:02; **2**)<br>IRELMEE**RC** (HLA-A*24:02; **1**)	Luo et al. 2021
***LCLAT1-ERBB4* (Group 3)**	TFVVDRLR**EV**CAGTENKL	FVVDRLR**EV** (HLA-A*02:01; **22**)<br>RLR**EV**CAGT (HLA-A*02:01; **19**)<br>VDRLR**EV**CA (HLA-A*02:01; **7**)<br>VVDRLR**EV**C (HLA-A*02:01; **6**)<br>DRLR**EV**CAG (HLA-A*02:01; **6**)<br>**EV**CAGTENK (HLA-A*02:01; **5**)<br>LR**EV**CAGTE (HLA-A*02:01; **3**)<br>R**EV**CAGTEN (HLA-A*02:01; **2**)	Jones et al. 2012

***MLLT6-MRPL45* (Group 4)**	SSASISTT**QP**VLVTQSAA	SISTT**QP**VL (HLA-A*02:01; **21**)<br>ASISTT**QP**V (HLA-A*02:01; **16**)<br>STT**QP**VLVT (HLA-A*02:01; **15**)<br>TT**QP**VLVTQ (HLA-A*02:01; **14**)<br>ISTT**QP**VLV (HLA-A*02:01; **13**)<br>SASISTT**QP** (HLA-A*02:01; **8**)<br>**QP**VLVTQSA (HLA-A*02:01; **8**)<br>T**QP**VLVTQS (HLA-A*02:01; **5**)	Jones et al. 2012
***EPC2-GULP1* (WNT + SHH)**	TGMEKEEE**SA**EEITLTIG	**SA**EEITLTI (HLA-A*02:01; **20**)<br>GMEKEEE**SA** (HLA-A*02:01; **14**)<br>KEEE**SA**EEI (HLA-A*02:01; **12**)<br>EE**SA**EEITL (HLA-A*02:01; **9**)<br>E**SA**EEITLT (HLA-A*02:01; **9**)<br>MEKEEE**SA**E (HLA-A*02:01; **2**)<br>EKEEE**SA**EE (HLA-A*02:01; **2**)<br>EEE**SA**EEIT (HLA-A*02:01; **0**)	identified and analyzed in the present work

* AA-sequence translated from published DNA sequence and expanded based on cDNA/AA-sequence alignments from ensembl.org

** fusion only in Asian cohort; SYFPEITHI analyses with HLA-A*02:01 (most common HLA-haplotype in Caucasian population) and with HLA-A*24:02 (most common HLA-haplotype in Asian population)
